# Comparative Effect of Insulin Resistance Reduction and Hormonal Alterations on Type 2 Diabetes Remission After Bariatric Surgery

**DOI:** 10.3390/jcm13226998

**Published:** 2024-11-20

**Authors:** Ekaterina Shestakova, Iurii Stafeev, Yury Yashkov, Anatoly Yurasov, Alina Tomilova, Yelena Parfyonova, Marina Shestakova, Ivan Dedov

**Affiliations:** 1 Endocrinology Research Centre, 117292 Moscow, Russia; 2 National Medical Research Center for Cardiology, 121552 Moscow, Russia; 3 Center of Endosurgery and Lithotripsy, 111123 Moscow, Russia; 4 Faculty of Medicine, M.V. Lomonosov Moscow State University, 117192 Moscow, Russia

**Keywords:** type 2 diabetes, obesity, remission, bariatric surgery, insulin, glucagon, GLP-1, GIP, incretin, insulin resistance

## Abstract

**Background:** Bariatric surgery is known to induce weight loss and diabetes remission in patients with type 2 diabetes (T2D), but the exact mechanism of glycemic normalization needs to be defined. **Methods:** The study included patients with BMI ≥ 35 kg/m^2^, obesity history ≥ 10 years, and planned bariatric surgery. At baseline and 3 and 6 months after surgery, all patients underwent anthropometric measurements, body composition and blood tests (including insulin, glucagon, and incretins during oral glucose tolerance test (OGTT)), and hyperinsulinemic euglycemic clamp tests. Diabetes remission was defined if the person reached HbA1c < 6.5% after surgery and glucose-lowering therapy withdrawal. **Results:** The study included 86 patients, divided into groups with no diabetes (control group, *n* = 44) and T2D (*n* = 42). Most patients with T2D reached normoglycemia at 6 months. BMI and insulin resistance (according to M-index) decreased in T2D group comparably to people without diabetes. At 6 months, people with T2D at baseline had less insulin and GLP-1 secretion and higher glucagon level during OGTT when compared to the control group. **Conclusions:** We conclude that weight and insulin resistance reduction is sufficient for T2D remission. The absence of insulin, glucagon, and incretin restoration is not crucial for the glucose metabolism in the short-term, but it may explain the relapse of T2D years after bariatric surgery.

## 1. Introduction

Weight loss is an important goal that patients with type 2 diabetes (T2D) need to achieve to improve glycemic control [1]. Among many strategies that may be used to induce weight loss, bariatric surgery is the most effective. After surgery, glycemia usually improves long before significant weight loss, which is why the exact explanation of how bariatric procedures cause T2D remission is still under debate. According to recent studies, the rate of diabetes remission may be as high as 72.3–100% two years after surgery, but decreases down to 25–60% by 10–15 years, depending on the type of bariatric procedure [2,3]. The decline in the diabetes remission rate is observed despite the sustained weight loss caused by bariatric surgery. Therefore, some controversial questions about surgery-induced T2D remission arise, such as the explanation of quick glycemia control soon after the bariatric procedure or the cause of T2D relapse years after surgery in spite of weight loss maintenance.

This study aimed to find out the leading reason for diabetes remission after bariatric surgery. Our hypothesis was that the main drivers of diabetes remission are the same as the drivers of diabetes development: the first is insulin resistance (IR) and the second is hormonal impairment. We investigated which one of these two factors is crucial for diabetes remission.

## 2. Materials and Methods

### 2.1. Subjects

Patients with obesity, with or without T2D, were eligible for inclusion according to the following criteria: age ≥ 30 years, body mass index (BMI) ≥ 35 kg/m^2^, history of being overweight or obese ≥ 10 years (overweight when BMI reached ≥ 25 kg/m^2^, obese if BMI ≥ 30 kg/m^2^), and planned bariatric surgery (sleeve gastrectomy/gastric bypass/single-anastomosis duodeno-ileal bypass with sleeve gastrectomy (SADI-S)). Bariatric surgery details have been described previously [4].

At screening, any glucose-lowering background therapy should have been stable for at least 8 weeks. Exclusion criteria were the following: type 1 diabetes, hemoglobin <70 g/L, estimated glomerular filtration rate (eGFR) < 60 mL/min/1.73 m^2^, baseline treatment with glucagon-like peptide 1 (GLP-1) receptor agonists, non-fatal myocardial infarction/non-fatal stroke 1 month prior to inclusion, history of abdominal surgery, pregnancy, and malignancy.

History of hypertension, coronary heart disease (CHD), obstructive sleep apnea, cholelithiasis, peptic ulcer disease, and smoking history were documented.

Eligible patients were examined at baseline and 3 and 6 months after surgery. The examination included anthropometry (height, weight, waist and hips circumference), body composition, blood tests, and a hyperinsulinemic euglycemic clamp test.

In patients with T2D, usual glucose-lowering therapy could be withdrawn no sooner than 3 months after surgery if the person reached HbA1c < 6.5%. Patients were recommended to maintain blood self-control (at least 4 daily fingerstick glucose measurements) and to contact the investigation center in case of stable blood sugar elevations.

### 2.2. Body Composition Analysis

The amounts of total and visceral fat were assessed by bioelectrical impedance analysis after overnight fasting using the Body Composition Analyzer Tanita MC-780MA (TANITA Corp., Tokyo, Japan). The analyzer calculated the visceral index (from the 1st to the 55th level) as an estimate of the amount of visceral adipose tissue and the total amount of body fat as a percentage of total body weight.

### 2.3. Glucose Tolerance Test and Mixed Meal Tests

An oral glucose tolerance test (OGTT) was performed in patients without known glucose disturbances, while patients with T2D received a mixed meal tolerance test (MMTT). Tests were conducted after overnight fasting and 24 h of antidiabetic drug deprivation. The blood samples for glucose, insulin, glucagon, and incretin hormones (GLP-1 and glucose-dependent insulinotropic peptide (GIP)) were collected at baseline and 30 and 120 min after a 75 g glucose load in the case of OGTT, or Oral Impact mix (Nestle Health Science, Vevey, Switzerland; 341 kcal, 9.2 g fat, 44.8 g carbohydrates, 18 g proteins) in the case of MMTT. The area under the curve (AUC) was calculated for glucose and hormones. Basal insulin secretion (HOMA-%B) was assessed by the following formula:

20 × fasting insulin (mU/mL)/fasting glucose − 3.5 (mmol/L)

The insulinogenic index, which represents the first phase of insulin secretion, was calculated as the ratio of insulin increment to glucose increment during OGTT/MMTT:

Insulin 30′ − fasting insulin (mU/mL)/glucose 30′ − fasting glucose (mmol/L)

### 2.4. Insulin Sensitivity Analysis

Systemic insulin resistance (IR) was measured by the classic R. DeFronzo hyperinsulinemic euglycemic clamp test [5] and by HOMA-IR, which was calculated as follows:

HOMA-IR = fasting insulin (mU/mL) ∗ fasting glucose (mmol/L)/22.5

For the clamp test insulin solution, 100 mU/mL was intravenously infused with constitutive rate 1 mU/kg/min using a compact syringe pump. Simultaneously a 20% glucose solution was also infused intravenously to reach normal blood glucose level, which was controlled every 5 min using the OneTouch “VerioPro” glucometer (Zug, Switzerland). The target blood glucose level was in the range 5.1–5.6 mmol/L. The dynamic equilibrium of the blood glucose level was achieved after 120–180 min of infusion, and at this moment the glucose infusion rate was assumed to be equal to glucose uptake by tissues. When the glucose infusion rate and blood glucose level reached the steady state, the M-value was calculated. The M-index is the expression of insulin resistance (IR) determined by the clamp test and is calculated as an arithmetic mean of 6–8 discrete values of the glucose infusion rate during 30–40 min of equilibrium divided by body weight. Thus, the M-index reflects the amount of glucose absorbed by one kilogram of the patient’s body per minute (mg/kg per minute). The results were expressed as M-values (mg/kg∗min) and classified into 4 groups of M-values: 0–2 (severe IR), 2–4 (moderate IR), 4–6 (mild IR), and >6 (no IR).

### 2.5. Laboratory Assays

HbA1c (reference values 4–6%) was assessed by high-performance liquid chromatography (D10 Hemoglobin Testing System, Bio-Rad, Hercules, CA, USA). Fasting blood glucose (FBG) (fasting reference values 3.1–6.1 mM) was assessed by the ARCHITECT c4000 Clinical Chemistry Analyzer (Abbott Diagnostics, Abbott Park, IL, USA) with manufacturer kits. Immune-reactive insulin was measured in serum with standard kit using the electrochemiluminescence analyzer Cobas 6000 (Roche, Basel, Switzerland). ELISA kits for glucagon and GLP-1 were obtained from Mercodia (Uppsala, Sweden); for GIP, from Cloud-Clone Corp. (Katy, TX, USA). The ELISA measurements were performed using the 1420 Multilabel Counter VICTOR2 (PerkinElmer, Shelton, CT, USA).

### 2.6. Statistical Analysis

The data were analyzed using Statistica 13.3 (TIBCO, Santa Clara, CA, USA). The data are presented as the median and interquartile range ([Q1; Q3] or 25–75%). Statistically significant differences between the control and T2D groups were evaluated by the Mann–Whitney rank sum U-test. Statistically significant differences in dependent values within one group were evaluated by Friedman ANOVA; between two time periods, by Wilcoxon test. *p*-values < 0.001 were considered significant.

## 3. Results

### 3.1. Baseline Parameters

Eighty-six patients with long histories (>10-years) of being overweight or obese were enrolled in the study and divided into groups with no previous hyperglycemia (control group, *n* = 44) and T2D (*n* = 42). The baseline characteristics are presented in Table 1.

The two groups were comparable according to most parameters, except for the HbA1c and triglyceride levels.

The groups did not differ according to the history of concomitant conditions except for hypertension, which was more prevalent in the T2D group (81.8% vs. 55% in the control group, *p* < 0.0002).

### 3.2. Metabolic Changes After Surgery

**Figure 1:**
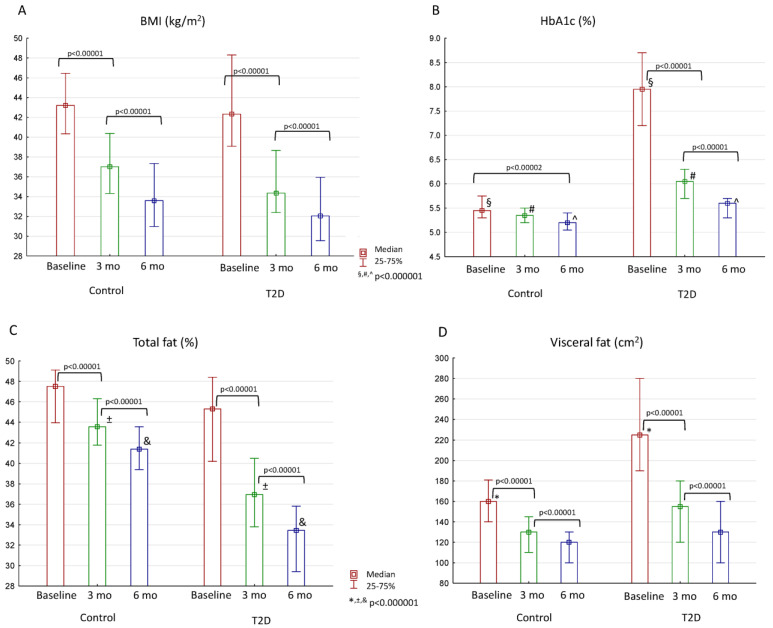
Changes in BMI, HbA1c, and fat distribution after bariatric surgery in control group and patients with T2D: (**A**)—change in BMI, (**B**)—change in HbA1c, (**C**)—change in amount of total fat, (**D**)—change in amount of visceral fat. Differences between the control and T2D groups are marked with special symbols (U-test), and changes between time periods within one group are marked with brackets (Wilcoxon test).

After bariatric surgery, both groups demonstrated weight and visceral fat reduction, as well as glycemic and lipid improvements (Figure 1A–D, Table 2). Total and visceral fat were assessed in bioelectrical impedance analysis.

Most of the patients in the T2D group reached normoglycemia 6 months after surgery: HbA1c in this group decreased from 8.0% [7.2; 8.7] to 6.1% [5.7; 6.3] in 3 months and 5.6% [5.3; 5.7] in 6 months. By the end of the study, the control group reached HbA1c of 5.2% [5.1; 5.4]. BMI did not differ between the groups in all study periods and dropped to 32.1 kg/m2 [29.5; 36.0] in the T2D group and to 33.6 kg/m^2^ [31.0; 37.3] in the control group. There was also no difference in the level of visceral fat.

The changes in waist and hip measurements and lipid profile are presented in Table 2.

The reduction of HbA1c was accompanied by antidiabetic drug withdrawal. The rate of drug deprescription is shown in Table 3. After surgery, antidiabetic drugs were stopped in 50% and 73.8% of patients at 3 and 6 months, respectively.

### 3.3. Insulin Resistance Changes After Surgery

**Figure 2:**
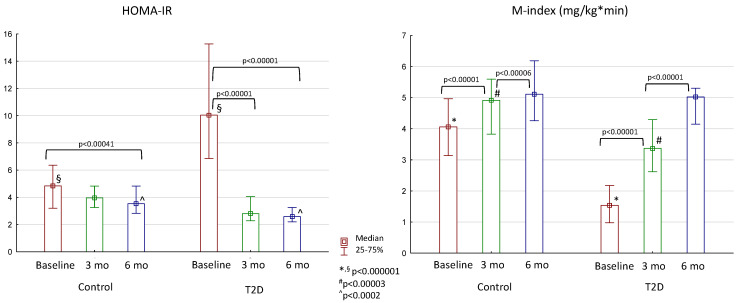
Changes in insulin resistance after bariatric surgery in the control group and patients with T2D. Differences between the control and T2D groups are marked with special symbols (U-test), and changes between time periods within one group are marked with brackets (Wilcoxon test).

According to the measurements of insulin resistance—hyperinsulinemic euglycemic clamp test and HOMA-IR—both groups showed improved insulin sensitivity throughout the study (Figure 2). The important observation is that the M-index in both groups became comparable at 6 months of follow-up, meaning that the patients with and without T2D had similar levels of insulin sensitivity at that time point.

### 3.4. Hormonal Changes After Surgery

**Figure 3:**
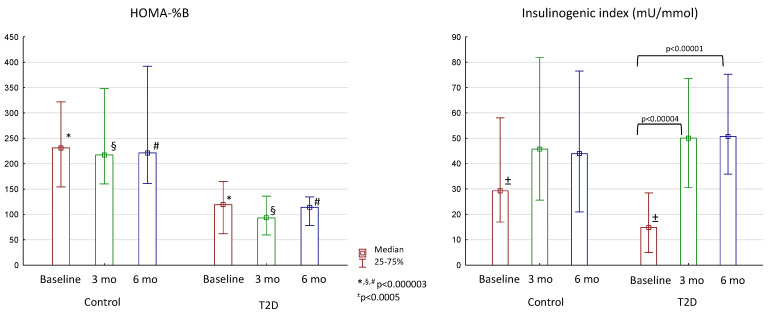
Changes in insulin secretion after bariatric surgery in the control group and patients with T2D. Differences between the control and T2D groups are marked with special symbols (U-test), and changes between time periods within one group are marked with brackets (Wilcoxon test).

We also assessed the secretion of insulin, glucagon, and main incretin hormones (GLP-1 and GIP) during OGTT/MMTT. While the insulinogenic index (first phase of insulin secretion) improved in T2D patients, basal insulin secretion, assessed by HOMA-%B, did not change after surgery and remained lower throughout the whole study when compared to the control group (Figure 3).

**Figure 4:**
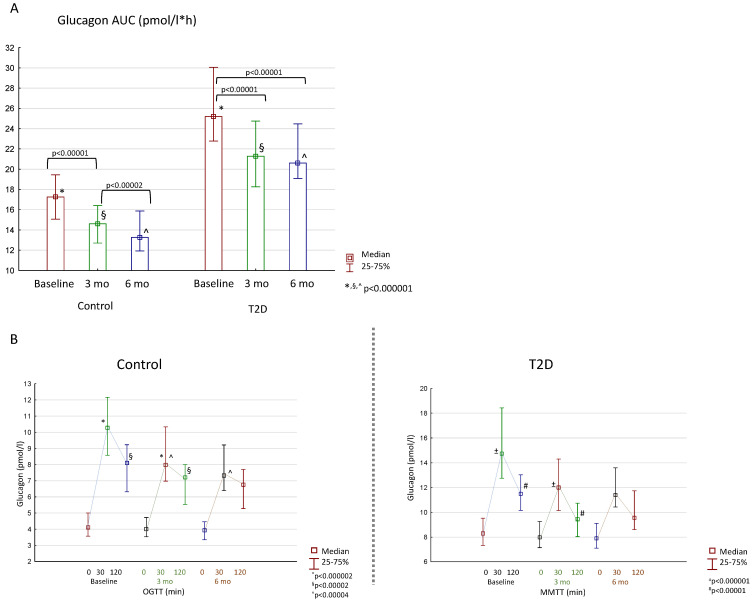
Changes in glucagon secretion after bariatric surgery in the control group and patients with T2D: (**A**)—change in glucagon AUC; (**B**)—pattern of glucagon secretion during OGTT/MMTT. Differences between the control and T2D groups are marked with special symbols (U-test), and changes between time periods within one group are marked with brackets (Wilcoxon test).

The total glucagon secretion was higher in the T2D group compared to the control not only before, but also 3 and 6 months after the bariatric surgery (Figure 4A). Notably, the pattern of glucagon secretion was similar in both groups regardless of the severity of glucose disturbance. The level of glucagon did not drop either after glucose loading in the control group or after mixed meal loading in the T2D patients (Figure 4B).

**Figure 5:**
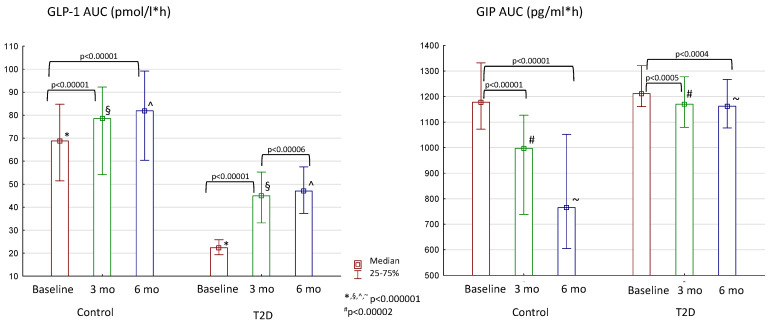
Changes in GLP-1 and GIP secretion after bariatric surgery in the control group and patients with T2D. Differences between the control and T2D groups are marked with special symbols (U-test), and changes between time periods within one group are marked with brackets (Wilcoxon test).

We also investigated how incretin hormones changed after meal loading in two groups. At baseline, patients with T2D had lower GLP-1 responses than the control group (Figure 5). The bariatric surgery led to improvements in GLP-1 secretion in both groups, but T2D patients still lacked GLP-1 compared to the control group. The opposite situation was observed with another incretin hormone—GIP. Its level was similar in patients with and without T2D at the beginning of the study. After bariatric surgery, GIP secretion decreased in both groups. At 6 months of the follow-up period, patients without T2D had much lower GIP levels than T2D patients.

Different types of operations could cause unequal changes in levels of pancreatic and gastrointestinal hormones; therefore, we conducted an additional analysis to compare hormonal secretion in patients who underwent various surgical interventions. There were no significant differences in glucagon, GLP-1, or GIP secretion depending on the type of surgery chosen, in patients both with and without T2D.

## 4. Discussion

The aim of the study was to understand whether decreases in weight and IR or hormonal (insulin, glucagon, and incretin) changes lead to diabetes remission after bariatric surgery. To implement this task, we included two cohorts of patients (with and without T2D), accurately matched by age, gender, BMI, WHR, and obesity history. We investigated how weight reduction, caused by bariatric surgery, influences two main pathogenetic mechanisms of diabetes development—insulin resistance and hormonal impairment.

In our study, patients in the two groups were carefully matched for most characteristics, including age, BMI, WC, and HC. They also had similar duration of obesity that reached nearly 20 years. The only two baseline parameters that differed in the groups were HbA1c and triglyceride levels. The difference in HbA1c was implied by the design of the study. As for the lipid profile, the differences were observed in the level of triglycerides, but not LDL, which may indicate the development of secondary hypertriglyceridemia in people with T2D. We previously showed that the fundamental base for additional triglyceride synthesis lays in interactions between visceral and subcutaneous fat depots [6].

That meant that each patient had a similar impact of excessive fat mass, which is a major risk factor for development of T2D. But despite very long histories of obesity and very high BMIs (above 40 kg/m^2^ in most participants), one group of patients developed T2D and the other group did not. We considered that the control group had some protective mechanisms that stopped them developing glucose intolerance. When we compared the results of bariatric surgery in both groups, we used the control group as a reference for the T2D group.

At baseline, individuals in the control group had a protective metabolic and hormonal phenotype: they had a less severe degree of IR according to the results of the “gold standard” definition of IR—the hyperinsulinemic euglycemic clamp test. These patients were characterized by a more favorable secretion profile: people without T2D had a higher peak insulin concentration and a lower degree of hyperglucagonemia, as well as a more preserved secretion of GLP-1. These results are consistent with previous observations: indeed, patients with T2D are deficient of insulin and GLP-1 secretion when compared to people with obesity but no glucose intolerance [7,8].

We were mostly interested in answering the question of whether bariatric surgery can reverse detrimental metabolic pathways in T2D. In other words, can this surgery lead to recovery of IR and hormonal disturbances to the same degree as in the control group?

Six months after bariatric surgery, patients with T2D were comparable to the control group according to the most parameters: BMI, visceral fat, and the level of IR, assessed by the hyperinsulinemic euglycemic clamp test. The reduction in visceral fat and IR led to significant improvements in glucose control in most of the patients. At least half of the patients reached diabetes remission, defined as a return of HbA1c to <6.5% that persists for ≥3 months in the absence of usual glucose-lowering pharmacotherapy [9]. At 6 months, 73.8% patients in the T2D group stopped the medication and achieved a median HbA1c of 5.6%. This meant that bariatric surgery was equally effective in reducing weight and IR in T2D patients as in the control group.

The second step was to determine whether bariatric surgery can restore insulin, glucagon, and incretin production in T2D patients. To understand this, we compared the level of hormonal secretion between T2D and controls at 6 months after surgery. At this time point, the T2D patients still had lower rates of basal insulin and GLP-1, as well as higher glucagon secretion, when compared to the control group. The difference between the groups persisted despite the improvements in insulin, glucagon, and GLP-1 production in T2D during the study. These results meant than hormonal restorations were not obligatory for diabetes remission after bariatric surgery.

The design of the study allowed us not only to compare the patients with and without T2D, but also to assess the sequence of pathophysiological changes in people with obesity after bariatric interventions. Follow-up examinations 3 and 6 months after the intervention were intended to identify the key factor for T2D remission: was it predominantly IR reduction or the change in hormone secretion? Unfortunately, we could not answer this question because all variables that could influence glycemia (body composition, IR, hormones) changed simultaneously during the first 3 months of follow-up, so we did not demonstrate the primary role of any of these factors in glycemic improvement in people with T2D.

Some important findings draw our attention. At first, postprandial hyperglucagonemia was detected in both groups at baseline and persisted throughout the study. The absence of postprandial glucagon suppression in people with T2D is established and is considered one of the pathogenetic mechanisms of hyperglycemia development [10,11]. However, the increase in glucagon levels after glucose loading in the control group (that consisted of patients with obesity without T2D) is not so obvious. Glucagon not only participates in the regulation of glucose metabolism, stimulating glycolysis and gluconeogenesis, but also causes a decrease in appetite and an increase in energy consumption [12,13]. It is possible that insufficient suppression of glucagon secretion occurs due to a decrease in GLP-1 level, which is an important regulator of glucagon in the postprandial period.

It is difficult to explain the high initial level of GIP in both groups. It remains unclear whether a high concentration of GIP in obese individuals can have a protective effect on the development of T2D. There is an uncertainty about the effects of GIP for therapeutic purposes: both stimulation of the receptors of this hormone and their blockade can have beneficial impact on body weight and insulin sensitivity [14,15,16]. It was found that GIP levels during OGTT gradually went up with increasing BMI [17]. At the same time, according to the study of the GLP-1/GIP co-agonist tirzepatide, stimulation of both incretin receptors has a bigger effect on reducing HbA1c and body weight compared to the GLP-1 receptor agonist semaglutide [18]. It is possible that the high basal level of GIP observed in our study may be explained by resistance to endogenous GIP in obesity.

The study has some limitations. Some of the T2D patients received glucose-lowering therapy at the end of the follow-up, which may have influenced the results of the laboratory examinations, though the patients stopped taking antidiabetic drugs 24 h before each visit. A total of 26.2% of patients in the T2D group were taking medications at the end of the study: most of them remained on metformin monotherapy, and two patients continued to receive a metformin + DPP-4 inhibitor combination. Metformin mainly affects IR, so those patients who received metformin could have had relatively low levels of IR compared to the patients who stopped all the drugs. However, according to the results of the study, HOMA-IR decreased significantly by the 3rd month of the follow-up and did not change by 6 months. At the same time, the number of patients receiving metformin 3 months after surgery was almost two times higher than the number of patients receiving the drug at 6 months. This may indicate a dominant contribution of weight loss, but not metformin therapy, to improved insulin sensitivity.

We also realize that the type of bariatric surgery could affect incretin secretion. Bypass procedures can induce a more significant increase in the level of GLP-1 compared to sleeve gastrectomy [19]; it is considered that this effect is due to faster food passage into the distal parts of the small intestine, although this may be controversial [20]. In our study, we performed additional analysis to compare hormonal secretion in groups of patients who underwent various surgical interventions. There were no significant differences in insulin, glucagon, GLP-1, or GIP, depending on the type of surgery, either in the control group or in patients with T2D.

Finally, we focused on the interaction between IR and hormonal secretion as the drivers for diabetes control after bariatric surgery but could not assess many additional factors that also affect glycemia in T2D patients. These factors include malabsorption, altered gut microbiota, cytokine production, etc.

## 5. Conclusions

Understanding why some patients with severe obesity do not develop hyperglycemia could help to find ways to prevent T2D. From a practical point of view, it is useful to identify the features of protective phenotypes of obesity. In our study, these were relatively low IR accompanied by high insulin and GLP-1 production as well as a low degree of hyperglucagonemia.

But we also aimed to answer the question of whether key pathogenetic mechanisms of T2D development could be restored after bariatric surgery in patients with long-term obesity (>10 years). Patients with previous T2D reached normoglycemia at 6 months, and their BMI and IR decreased comparably to people without diabetes, but hormonal restoration did not occur. We conclude that weight and IR reduction may be sufficient for diabetes remission after bariatric surgery, but the lack of secretion of insulin and GLP-1, as well as glucagon excess, may cause further glycemic deterioration in T2D patients. These results may indicate that people with diabetes need to continue long-term glycemic control after surgery.

## Figures and Tables

**Table 1:** Baseline anthropometric and clinical characteristics.

	Control (*n* = 44)	T2D (*n* = 42)	*p* (U-Test)
Gender (m/f) *n*/%	10/34 (23/77%)	17/25 (40/60%)	0.076 *
Age (years)	45.5 [40.5; 49]	48.5 [42; 54]	0.019
BMI (kg/m^2^)	43.2 [40.3; 46.5]	42.3 [39.1; 48.3]	0.826
Waist circumference (WC, cm)	133 [128; 140]	128 [121; 137]	0.042
Hips circumference (HC, cm)	128.5 [118; 136]	127 [115; 132]	0.201
WHR	1.05 [0.99; 1.12]	1.05 [0.99; 1.09]	0.782
HbA1c (%)	5.5 [5.3; 5.8]	8.0 [7.2; 8.7]	<0.0003
Total cholesterol (mmol/L)	5.4 [4.7; 5.8]	5.3 [4.5; 6.1]	0.839
LDL (mmol/L)	3.3 [2.9; 3.7]	3.4 [2.9; 4.1]	0.278
HDL (mmol/L)	1.1 [1.0; 1.5]	1.1 [1.0; 1.3]	0.397
Triglycerides (mmol/L)	1.4 [1.0; 2.0]	2.6 [1.7; 3.4]	<0.0003
Obesity history (years)	18 [15; 25]	20 [15; 27]	0.120
T2D history (years)	-	9 [6; 12]	-

Data presented as median [Q1; Q3]. WHR—waist to hip ratio, LDL—low-density lipoprotein, HDL—high-density lipoprotein, * chi-squared test.

**Table 2:** Changes in waist, hip circumference, and lipid profile.

	Baseline	3 Months	6 Months	*p* (Friedman ANOVA)
Control group				
	*n* = 44	*n* = 44	*n* = 44	
Waist circumference (WC, cm)	133 [128; 140]	122 [117.5; 130]	115 [110; 120]	<0.00001
Hips circumference (HC, cm)	128.5 [118; 136]	119.5 [109; 126]	109 [100.5; 114]	<0.00001
WHR	1.05 [0.99; 1.12]	1.04 [1.0; 1.09]	1.07 [1.03; 1.11]	0.002
Total cholesterol (mmol/L)	5.4 [4.7; 5.8]	5.0 [4.3; 5.3]	4.8 [4.3; 5.1]	<0.0002
LDL (mmol/L)	3.3 [2.9; 3.7]	3.1 [2.6; 3.5]	3.0 [2.5; 3.4]	<0.00001
HDL (mmol/L)	1.1 [1.0; 1.5]	1.4 [1.2; 1.7]	1.6 [1.3; 2.0]	<0.00001
Triglycerides (mmol/L)	1.4 [1.0; 2.0]	1.2 [0.9; 1.8]	1.1 [0.9; 1.7]	<0.00001
T2D group				
	*n* = 42	*n* = 42	*n* = 42	
Waist circumference (WC, cm)	128 [121; 137]	109.5 [105; 120]	105 [101; 115]	<0.00001
Hips circumference (HC, cm)	127 [115; 132]	116 [108; 128]	113 [104; 123]	<0.00001
WHR	1.05 [0.99; 1.09]	0.96 [0.93; 1.0]	0.96 [0.92; 0.98]	<0.00002
Total cholesterol (mmol/L)	5.3 [4.5; 6.1]	4.5 [3.7; 5.0]	4.2 [3.7; 4.8]	<0.00001
LDL (mmol/L)	3.4 [2.9; 4.1]	3.1 [2.7; 3.7]	3.1 [2.6; 3.6]	<0.00001
HDL (mmol/L)	1.1 [1.0; 1.3]	1.2 [1.0; 1.4]	1.3 [1.2; 1.5]	<0.00001
Triglycerides (mmol/L)	2.6 [1.7; 3.4]	2.0 [1.3; 2.4]	1.8 [1.3; 2.2]	<0.00001

**Table 3:** Number and classes of antidiabetic drugs in T2D group.

	Baseline	3 Months	6 Months
Number of T2D patients taking antidiabetic drugs (% of total group)			
Monotherapy	11 (26.2%)	17 (40.5%)	9 (21.4%)
Dual therapy	19 (45.2%)	4 (9.5%)	2 (4.8%)
incl. on insulin	1	0	0
Triple therapy	12 (28.6%)	0	0
incl. on insulin	5	0	0
Classes of antidiabetic drugs (*n*.% of total group)			
Metformin	33 (78.6%)	20 (47.6%)	11 (26.2%)
DPP-4 inhibitors	17 (40.5%)	3 (7.1%)	2 (4.8%)
SGLT-2 inhibitors	13 (31%)	2 (4.8%)	0
SUs	16 (38.1%)	0	0
Basal insulin	6 (14.3%)	0	0

DPP-4—dipeptidyl peptidase-4, SGLT-2—sodium-glucose co-transporter 2, SUs—sulfonylureas.

## Data Availability

Data are contained within the article.
